# Study on the mechanism of MDSC-platelets and their role in the breast cancer microenvironment

**DOI:** 10.3389/fcell.2024.1310442

**Published:** 2024-02-09

**Authors:** Xinpu Han, Xiaotong Song, Zhigang Xiao, Guanghui Zhu, Ruike Gao, Baoyi Ni, Jie Li

**Affiliations:** ^1^ Department of Oncology, Guang’anmen Hospital, China Academy of Chinese Medical Sciences, Beijing, China; ^2^ Department of Hematology-Oncology, Dongzhimen Hospital, Beijing University of Chinese Medicine, Beijing, China; ^3^ Department of Oncology, First Hospital of Heilongjiang University of Chinese Medicine, Heilongjiang University of Chinese Medicine, Harbin, China

**Keywords:** breast cancer, microenvironment, MDSCs, platelets, mechanisms

## Abstract

Myeloid-derived suppressor cells (MDSCs) are key immunosuppressive cells in the tumor microenvironment (TME) that play critical roles in promoting tumor growth and metastasis. Tumor-associated platelets (TAPs) help cancer cells evade the immune system and promote metastasis. In this paper, we describe the interaction between MDSCs and TAPs, including their generation, secretion, activation, and recruitment, as well as the effects of MDSCs and platelets on the generation and changes in the immune, metabolic, and angiogenic breast cancer (BC) microenvironments. In addition, we summarize preclinical and clinical studies, traditional Chinese medicine (TCM) therapeutic approaches, and new technologies related to targeting and preventing MDSCs from interacting with TAPs to modulate the BC TME, discuss the potential mechanisms, and provide perspectives for future development. The therapeutic strategies discussed in this review may have implications in promoting the normalization of the BC TME, reducing primary tumor growth and distant lung metastasis, and improving the efficiency of anti-tumor therapy, thereby improving the overall survival (OS) and progression-free survival (PFS) of patients. However, despite the significant advances in understanding these mechanisms and therapeutic strategies, the complexity and heterogeneity of MDSCs and side effects of antiplatelet agents remain challenging. This requires further investigation in future prospective cohort studies.

## 1 Introduction

In 2020, the International Agency for Research on Cancer (GLOBOCAN) estimated that, among 36 cancers in 185 countries worldwide, breast cancer (BC) has surpassed lung cancer as the most prevalent malignancy and is ranked as the fifth-leading cause of all cancer-related deaths. In 2020, there were over 2.3 million new cases and 685,000 fatal cases of BC worldwide (Sung et al., 2021), accounting for approximately 24.5% of all female cancer cases and 15.5% of all cancer-related deaths. BC is a leading cause of morbidity and mortality in most countries around the globe (Sung et al., 2021), making it an urgent health concern. The number of new BC cases is expected to reach 4.4 million by 2070 (Soerjomataram and Bray, 2021). The main risk factors for BC are reproductive factors (late-onset menopause, early-onset menarche, first birth after 30 years of age, short breastfeeding duration, and childlessness), familial genetics, lifestyle (tobacco exposure, chronic alcohol consumption, physical inactivity, high-fat diets, low vitamin D intake, and short sleep duration), environmental factors (chronic stress, poor mood, and exposure to excessive ionizing radiation), and other factors (obesity, other breast diseases, use of hormone therapy or oral contraceptives during menopause) (Britt et al., 2020; Zhang et al., 2020).

In recent years, it has been demonstrated that BC includes not only tumor cells, but also significant alterations in the tumor microenvironment (TME) or surrounding stroma. These alterations, such as myeloid-derived suppressor cell (MDSC) interactions with platelets, and alterations in the immunosuppressive, metabolic, and angiogenic microenvironments, can hinder effective anti-tumor immunity and promote BC progression and metastasis. These alterations are now recognized as key factors in BC progression and may be potential therapeutic targets. Based on the expression of three important BC markers, namely, the progesterone receptor, estrogen receptor, and human epidermal growth factor receptor 2, BC can be categorized into four types: luminal A, luminal B, HER2 (+), and triple-negative breast cancers. BC is based on these classifications, along with the main treatments, such as surgery, radiotherapy, chemotherapy, targeted therapy methods, and endocrine therapy (Hortobagyi, 1998). However, the complex TME scenario does not consider the classification-based solution, which has been shown to compromise the efficacy (Sharma et al., 2010; Łukasiewicz et al., 2021).

Therefore, characterizing the interactions between cancerous and noncancerous cells in the TME may reveal the critical vulnerabilities of BC and provide new diagnostic and therapeutic perspectives. Several new therapies that target the microenvironmental and stromal components have been developed for clinical trials. In this study, we review the specific interactions between MDSCs, platelets, and BC cells that modulate the BC microenvironment and summarize the currently investigated drugs, therapeutic strategies, and new technologies.

## 2 MDSCs: definition and role during tumor progression

### 2.1 Definition and generation of MDSCs

The tumor immune microenvironment is crucial for the development, growth, and treatment of tumors (Robert et al., 2015). Bone marrow cells are the most abundant hematopoietic cells in the immune system and have multiple physiological and pathological functions (Gabrilovich et al., 2012). Myeloid progenitor cells and immature myeloid cells are unable to differentiate into mature myeloid cells under pathological conditions, such as continuous inflammatory stimulation or tumors. This leading to the growth of pathogenic myeloid cells and cells with immunosuppressive properties, which accumulate in large numbers in the organism to form a heterogeneous population known as MDSCs. MDSCs are characterized by a morphological mix of granulocytes and monocytes resulting from a mixture of dendritic cells (DCs), macrophages, and granulocytic precursors (Pillay et al., 2013), which can significantly inhibit immune cell responses, promote the immune escape of tumor cells (Marvel and Gabrilovich, 2015) and diminish the effectiveness of cancer immunotherapy.

In 1929, cancer was first associated with abnormal myelopoiesis (Sonnenfeld, 1929). MDSCs are absent in healthy humans or mice. A systemic increase in immature myeloid cells was first identified in mice with immunosuppressive tumors in the late 1970s (Lee and Rosse, 1982); however, it was not until 1996 that this phenomenon was first observed in humans (Talmadge et al., 1996), the term MDSCs was confirmed in 2007 (Gabrilovich et al., 2007). MDSCs are divided into two primary subtypes: monocytic MDSCs (M-MDSCs) and granulocyte-MDSC (G-MDSCs, also known as polymorphonuclear MDSCs [PMN]-MDSCs). The surface phenotypes of MDSCs vary considerably between mice and humans. In mice, MDSCs express cell surface molecular markers, such as CD11b+Gr-1+. Depending on the expression and morphology of the Gr-1 epitope-specific antibodies Ly6G and Ly6C, G-MDSCs (PMN-MDSCs) are described as CD11b+Ly6G + Ly6C low, and M-MDSCs are described as CD11b+Ly6G- Ly6C high (Sinha et al., 2008). However, in cancer patients, there are three main subgroups of MDSC, PMN-MDSC, M-MDSC, and early stage MDSCs (e-MDSCs). PMN-MDSC is defined as CD14^-^/CD66b^+^/CD33^dim^/HLA-DR^low^ or HLA-DR^negative^, M-MDSC is defined as CD14^+^/CD66b^−^/CD33^high^/HLA-DR^low^, and e-MDSC defined as CD14^-^/CD66b^−^/CD33^dim^/HLA-DR^low^ or HLA-DR^negative^ (Lang et al., 2018). The PMN-MDSCs of the double-positive subset of CD11b and CD16 exhibited the strongest inhibition of T-cell activity and were the most potent T-cell suppressive MDSC subset, and high frequencies of PMN-MDSC in circulation were strongly correlated with low patient survival. m-MDSC also exhibited substantial inhibition of T-cell function, and high frequencies of this subset were similarly associated with poor outcomes. e-MDSC had no T-cell inhibitory effect and there was no correlation with overall survival. Thus, the emerging clinical relevance of PMN-MDSC and M-MDSC in human oncology should be focused on (Shipp et al., 2016).

### 2.2 Role of MDSCs in tumor development

MDSCs are among the most effective immunosuppressive cell types, with great potential to inhibit immune responses *in vitro* and *in vivo*. MDSCs, which are rare in healthy humans, are significantly increased in the peripheral blood of patients with tumors. This is conducive to the formation of microenvironments suitable for tumor development, promotion of tumorigenesis and metastasis, and the generation of immune tolerance. Once MDSCs migrate to the TME, they assume consume important trophic elements necessary for the development of T cells and natural killer (NK) cells through the generation of reactive oxygen species (ROS) and nitric oxide (NO), thereby inhibiting their proliferation, development, and cytotoxicity (Zhang et al., 2015). They may also hinder the capacity of B cells to present antigens and promote the growth of regulatory T cells (Tregs) to be recruited into the TME. This may also induce Tregs to exert a sustained immune-suppressant effect, aggravate the tumor site’s local immunity malfunction, and promote the immune escape of tumor cells (Groth et al., 2019).

Tumor microvessels tend to be more tortuous and permeable than those in healthy tissues. These effects are largely attributed to the ability of MDSCs to increase vascular endothelial growth factor (VEGF) expression through transforming growth factor (TGF)-β signaling (Bordeleau et al., 2017). MDSC-produced matrix metalloproteinase 9 (MMP9) disrupts vascular integrity by sequestering vascular TGF-β and VEGF in the extracellular matrix (ECM) (Ebrahem et al., 2010), leading to vascular leakage as well as collagen reorganization in the basement membranes of the pre-metastatic pulmonary vasculature (Tjiu et al., 2009). This promotes ECM remodeling and pre-metastatic microenvironment (PMN) formation of tumors (Ahn and Brown, 2008), which enhances the ability to promote the seeding of circulating tumor cells (CTCs) in the lungs (Kim et al., 2009). Yan et al. (Yan et al., 2010) found that BC cells exhibited a significant increase in Gr-1+CD11b+ cells prior to reaching the mouse lungs and produced large amounts of MMP9 to promote vascular remodeling and transforming the pre-metastatic lungs into a proliferative environment. In contrast, MMP9 deletion normalized abnormal pre-metastatic lung vasculature and reduced lung metastasis.

## 3 Role of platelets and tumor-associated platelets (TAPs) in tumor progression

### 3.1 Platelets are a double-edged sword

Platelets and their granular contents can directly or indirectly maintain the anticoagulant and procoagulant balance and modulate inflammatory and immune responses. However, platelet activation in the absence of vascular injury can render platelets to become a double-edged sword. Platelets fill the void that is created when the vessel wall is damaged, contributing to the integrity of the vessel wall (Repsold and Joubert, 2021). In addition to playing a crucial function in hemostasis, platelets also play a role in coordinating immunological and inflammatory responses through interactions with leukocytes and endothelial cells (ECs) as well as the release of soluble inflammatory mediators that promote leukocyte recruitment and activation (Karshovska et al., 2013). Viral infection activates platelets to promote the release of *ß*-defensins, which have been reported to destroy a variety of viruses (Wilson et al., 2013). In hepatitis B virus-infected mice, platelets are responsible for the intrahepatic accumulation of virus-specific cytotoxic T lymphocytes (Iannacone et al., 2005) and P-selectin (PS) mediates the entry of cytotoxic T lymphocytes into hepatic tissues, which increases the removal of viruses and enhances tissue damage (Iannacone et al., 2007).

Unnecessary platelet activation is a response to internal damage, platelet activation has also been shown to interact with tumor dynamics to promote tumor cell proliferation and metastasis (Tesfamariam, 2016). Platelet activation and increased numbers are linked to an elevated risk of thrombotic conditions. However, the risk of bleeding is also enhanced by thrombocytopenia and various platelet dysfunctions. For example, it can promote exfoliation and erosion of endothelial surfaces or the rupture of atherosclerotic plaques (Badimon et al., 1992), and is a major contributor to the emergence of sepsis-related complications, such as acute lung injury and acute renal injury (de Stoppelaar et al., 2014). Unstable thrombi tend to occlude small blood vessels and impair oxygen supply to target organs. Therefore, platelets are thought to be responsible for the fatal stages of Cerebrovascular Disease (Lebas et al., 2019). Thrombocytosis is a common finding in patients with cancer (10%–57%) (Sierko and Wojtukiewicz, 2004). For decades, an increased risk of thrombosis and platelet activation has been observed in patients with BC (Chew et al., 2006). Since 1968, the connection between platelets and BC metastasis has been well studied (Lal et al., 2013; Schlesinger, 2018). Importantly, platelet interaction with CTCs helps to protect the tumor cells from shear induced cell death and to shield them from immune responses (Xu et al., 2016; Morris et al., 2022). Strikingly, tumor cell induced platelet activation is a critical step to enable tumor cell extravasation and invasion of target tissues of metastasis (Li et al., 2021). Following experimental thrombocytopenia induced in animal trials using antiplatelet serum and neuraminidase, Gasic et al. discovered a 50% decrease in BC metastasis (Gasic et al., 1968). This antimetastatic effect was effectively reversed by the transfusion of platelet-rich plasma. These results indicate that platelets play a crucial role in cancer etiology (Sierko and Wojtukiewicz, 2004).

### 3.2 TAPs: definition and role in tumor progression

In the TME, normal platelets can be indirectly activated by Tissue Factor (TF), cancer procoagulant (CP), and collagen secreted by tumor cells through coagulation activation (Sierko and Wojtukiewicz, 2004). The TME can also be overactivated by binding of P-selectin glycoprotein ligand-1 (PSGL-1) to the surface of MDSCs interact with platelets via their expression of PS. Platelets with the ability to induce tumor cell invasion, metastasis, and angiogenesis are defined as TAPs. TAPs can infiltrate the tumor environment and play a more complex role, interacting with tumor cells through the direct binding of surface receptors and molecules or the secretion of cytokines (Zhang et al., 2018). On the one hand, factors released by TAPs can exhibit cytotoxic effects on proliferating tumor cells or even enhance apoptosis or induces a dormant state in the tumor cells (Dymicka-Piekarska et al., 2021). TAPs can also adhere to CTCs to form a physical barrier to protect them from NK cell invasion and tumor necrosis factor *a* (TNF-α)-mediated cytotoxicity, promoting the survival of tumor cells (Lal et al., 2013). On the other hand, TAPs also promote tumor metastasis by affecting the TME and promoting neovascularization (Riedl et al., 2014). A direct interaction between BC cells and platelets also activates the TGF-β signaling pathway, which inhibits NK cell activation or function by suppressing mammalian target of rapamycin (mTOR) activity (Xu et al., 2018), and induces epithelial-mesenchymal transition (EMT) and immunosuppression to promote invasion and metastasis (Drabsch and Ten Dijke, 2011). In addition to TGF-β, lactate released from TAPs may also inhibit T cell proliferation, interferon (IFN)-γ production, and embryogenesis (Wang et al., 2018), thereby creating a tumor immunosuppressive microenvironment.

## 4 Interaction of MDSCs with platelets contributes to BC development

### 4.1 Effect of MDSCs on platelets

MDSCs are closely associated with platelets and PSGL-1 on the surface of MDSCs binds to the platelet PS to activate platelets in TAPs via the PSGL1/PS pathway. MDSCs also play a significant role in platelet formation and recruitment, which cause malignant outcomes in patients with BC. Zhou et al. (Zhou et al., 2017) examined MDSCs and Tregs in peripheral blood samples from 25 patients with primary immune thrombocytopenia (ITP) and 10 healthy individuals. MDSCs were reduced in patients with ITP compared to the healthy individuals. In addition, patients with relapsed ITP had lower MDSC levels than those with new-onset ITP. This results indicated that a high number of MDSCs is not only related to the number of platelets but also to the severity of ITP. Hou et al. (Hou et al., 2016) administered high-dose dexamethasone (DXM) to patients with ITP and found that it significantly increased platelet levels and increased disease severity by upregulating MDSC expression. This may be because DXM-modulated MDSCs attenuate cytotoxic T lymphocyte-mediated platelet lysis. CXC chemokine ligand 17 (CXCL17), a novel CXC chemokine consisting of 119 amino acids, is negatively correlated with CD4^+^ T cell aggregation and promotes BC growth and metastasis through significant pro-angiogenic effects. The tumor immunosuppressive microenvironment is primarily caused by the infiltration of immunosuppressive cells (MDSCs). Platelet-derived growth factor-BB (PDGF-BB) is an oncogenic factor that is involved in various cancer metastases (Kumar et al., 2018). It was reported (Hsu et al., 2019) that CXCL17 secreted by primary BC can increase the accumulation of MDSCs (CD11b^+^Gr1^+^) in the lungs, resulting in the production of high levels of PDGF-BB, which leads to the enhancement of angiogenesis in lung tissues before the arrival of BC cells, laying the foundation for BC lung metastasis. In addition, chemokines released by MDSCs can accelerate TAPs recruitment to the TME (Lu et al., 2021).

### 4.2 Effect of platelets on MDSCs

**FIGURE 1:**
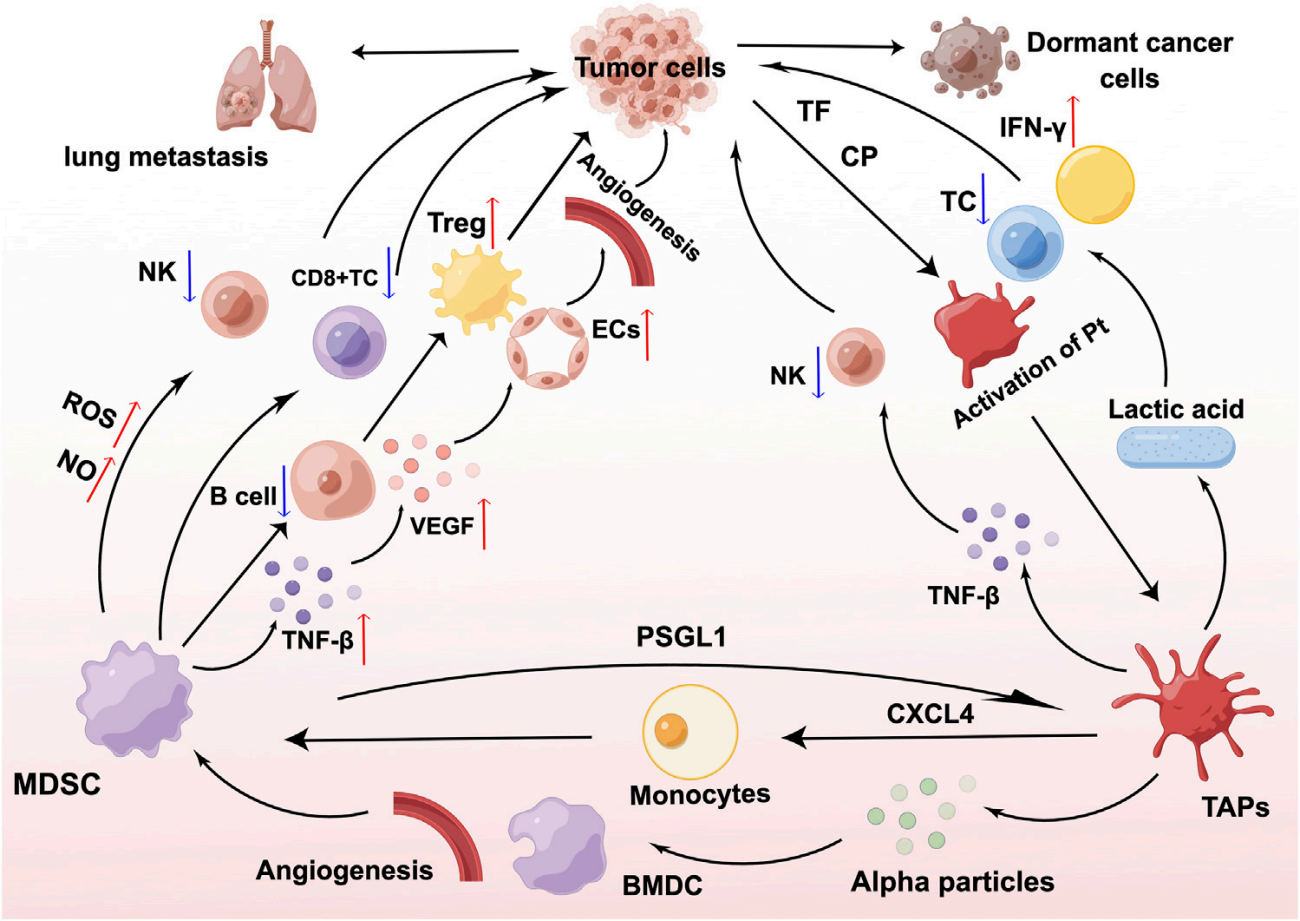
Interaction of myeloid-derived suppressor cells (MDSCs), platelets, and cancer cells. MDSCs inhibit the proliferation and development of T cells and natural killer (NK) cells through the production of nitric oxide (NO) and reactive oxygen species (ROS), interfere with the antigen-presenting ability of B cells, and stimulate the proliferation of regulatory T cells (Tregs) in large numbers, thereby exerting a sustained immunosuppressive effect. MDSCs derive transforming growth factor-β (TGF-β) and increase the expression of vascular endothelial growth factor (VEGF), which acts on endothelial cells (ECs) and leads to tumor microvascular malformations. TF and CP secreted by cancer cells as well as MDSCs are able to transform platelets to tumor-associated platelets (TAPs) through the PSGL1/P-selectin pathway. TAPs can drive tumor metastasis by promoting MDSCs recruitment, immunosuppression, and neovascularization. Figure was created by Figdraw (www.figdraw.com).

Platelets play an influential role in MDSCs, and can promote the generation and recruitment of MDSCs, and enhance the immunosuppressive activity of MDSCs, leading to malignant outcomes in patients with tumors. CD8^+^T cells inhibit tumor metastasis. According to Joseph et al. (Joseph et al., 2021), platelets release CXCL4 when the equilibrium between CD8^+^ T cells and platelets is disrupted, causing monocytes to develop into MDSCs. In turn, MDSCs inhibit the function of CD8^+^T cells and promote tumor metastasis. MDSCs are immature bone marrow-derived cells (BMDCs) that expand and infiltrate tumors in patients with malignancy. Platelets are capable of inducing BMDC recruitment as well as promoting tumor angiogenesis and growth through the secretion of *a*-granules (Feng et al., 2011), and to a certain extent, they can also promote the recruitment of MDSCs. Platelets are key players in inflammation and are closely associated with tumor development. A mouse model of colitis-associated cancer demonstrated that platelets promote MDSC recruitment and actively participate in colitis-induced tumor initiation (Ullman and Itzkowitz, 2011). Platelets also contribute to the promotion of myeloid cell polarization toward an inhibitory phenotype, and myeloid inhibitory cell expansion and phenotypic modifications associated with carcinogenesis are dependent on platelets. *In vivo* and *in vitro* experimental data from Servais et al. (Servais et al., 2018) revealed that platelets and platelet-derived soluble factors potentiate the inhibitory function of MDSCs at the transcriptional level. In in vivo experiments, the occurrence of G-MDSC-platelet aggregates in the circulation was linked to the development of cancer. In in vitro experiments, co-incubation of platelets or platelet releasates from tumor-bearing mice with MDSCs revealed that platelets were able to considerably increase the ability of MDSCs to block T cell proliferation and increase the levels of Arginase-1(Arg-1) and C/EBPβ mRNA expression, consequently encouraging tumor development by assisting in immune evasion (Servais et al., 2018). The ability of clopidogrel to suppress inflammation and prevent tumorigenesis by inhibiting the cancer-induced accumulation of MDSCs through its antiplatelet effect supports this concept (Servais et al., 2018). The interplay among MDSCs, platelets, and cancer cells is shown in Figure 1.

## 5 MDSCs and platelet-regulated BC TME

### 5.1 Immune microenvironment

The TME, which includes immune cells and immunomodulation-related cytokines, is a crucial element (Deshmukh et al., 2017). A large number of immunosuppressive cells, such as MDSCs, Tregs, tumor-associated macrophages (TAMs), and a large number of inflammation-related factors, such as interleukin (IL)-6, IL-10, TGF-β, etc., accumulate in large quantities in the TME, which together, promote tumor immune escape, tumor growth, and metastasis (Dunn et al., 2004). The state of the BC immune microenvironment is correlated with the prognosis of BC and the efficacy of immunotherapy.

MDSCs react directly with BC cells. Studies have shown that BC cells can recruit MDSCs through the STAT3-NF-kB-IDO, STAT3/IRF-8, and PTEN/AKT pathways, amongst others (Shou et al., 2016). MDSCs that converge at or toward the tumor site can act directly on tumor cells to induce EMT and promote BC metastasis by upregulating TGF31, VEGF, and IL-10 levels. In addition, MDSCs suppress immunological responses through a variety of mechanisms, and their immunosuppressive activity mainly targets T cells and NK cells. The two classical pathways by which MDSCs inhibit T cell anti-tumor immunity are: First, MDSCs that are activated by Th1 cytokines upregulate the expression of inducible nitric oxide synthase (iNOS), which induces NO production, inhibiting T cell responses. Second, MDSCs activated by Th2 cytokines upregulate the expression of Arg-1, which catalyzes the production of peroxynitrite from arginine and severely inhibits T cell activation (Gabrilovich and Nagaraj, 2009). At the same time, MDSCs induce the transformation of tumor-specific T cells into Tregs via mechanisms that are dependent on IL-10 and TGF-β. Safarzadeh et al. (Safarzadeh et al., 2019) reported an increase in the recruitment of MDSCs through the use of co-culture experiments of purified MDSCs (HLA-DR-CD33^+^). In these experiments, with CD3^+^T cells, and found that MDSCs and the inhibition of T cell proliferation was more potent and had a dual immunosuppressive effect in a group of patients with BC when compared to a healthy control group. MDSCs can mediate the immune response of NK cells by downregulating the expression of NK cell surface activation receptors, NKp30, NKG2D, and NKp46 (Han et al., 2018), and establish a pre-metastatic niche (PMN) through the promotion of angiogenesis and the recruitment of other immunosuppressive cells (Bruno et al., 2019). MDSCs also interact with other immune cells, including macrophages, Tregs, and DCs (Ostrand-Rosenberg et al., 2012). MDSCs can polarize macrophages toward the M2 phenotype, which promotes tumor progression, secrete IL-10 and TGF-β, induce the proliferation of Treg cells, and indirectly inhibit CD4^+^ Th1 and CD8^+^ T cell activity through the inhibition of DCs, exerting immunosuppressive functions (Yaseen et al., 2020).

Platelets are also closely involved in the functional role of NK cells. Platelets can promote the transition of tumor cells into a dormant state, resulting in the formation of dormant cancer cells (Dymicka-Piekarska et al., 2021). This not only reduces NK cells killing by forming a physical barrier on the surface of tumor cells but also disguises tumor cells as normal cells by transferring major histocompatibility complex (MHC) class I molecules to the surface of tumor cells, rendering them unrecognizable to NK cells (Placke et al., 2012). Thus, tumor cells evade NK cell-mediated immune attacks during PMN development (Garner and de Visser, 2020). TAPs that adhere to CTCs can prevent chemotherapeutic drugs from reaching cancer cells through physical barriers, making cancer cells less responsive to cytotoxic chemotherapeutic drugs that target actively proliferating cells. Both spontaneous and experimental metastases of tumor cells were significantly reduced in Galphaq (a G protein essential for platelet activation)-deficient mice, whereas this effect was not observed in NK cell-deficient mice, suggesting that activated platelets may reduce NK cells induced tumor cells killing by wrapping around tumor cells (Palumbo et al., 2005). *In vitro* findings support the *in vivo* observation that platelets aggregate around tumor cells to inhibit the killing activity of NK cells (Nieswandt et al., 1999). In addition, glucocorticoid-induced tumor necrosis factor receptor ligand (GITRL) on the platelet surface inhibits cytotoxicity and IFN-γ secretion from NK cells by activating GITR on NK cells (Placke et al., 2012).

**FIGURE 2:**
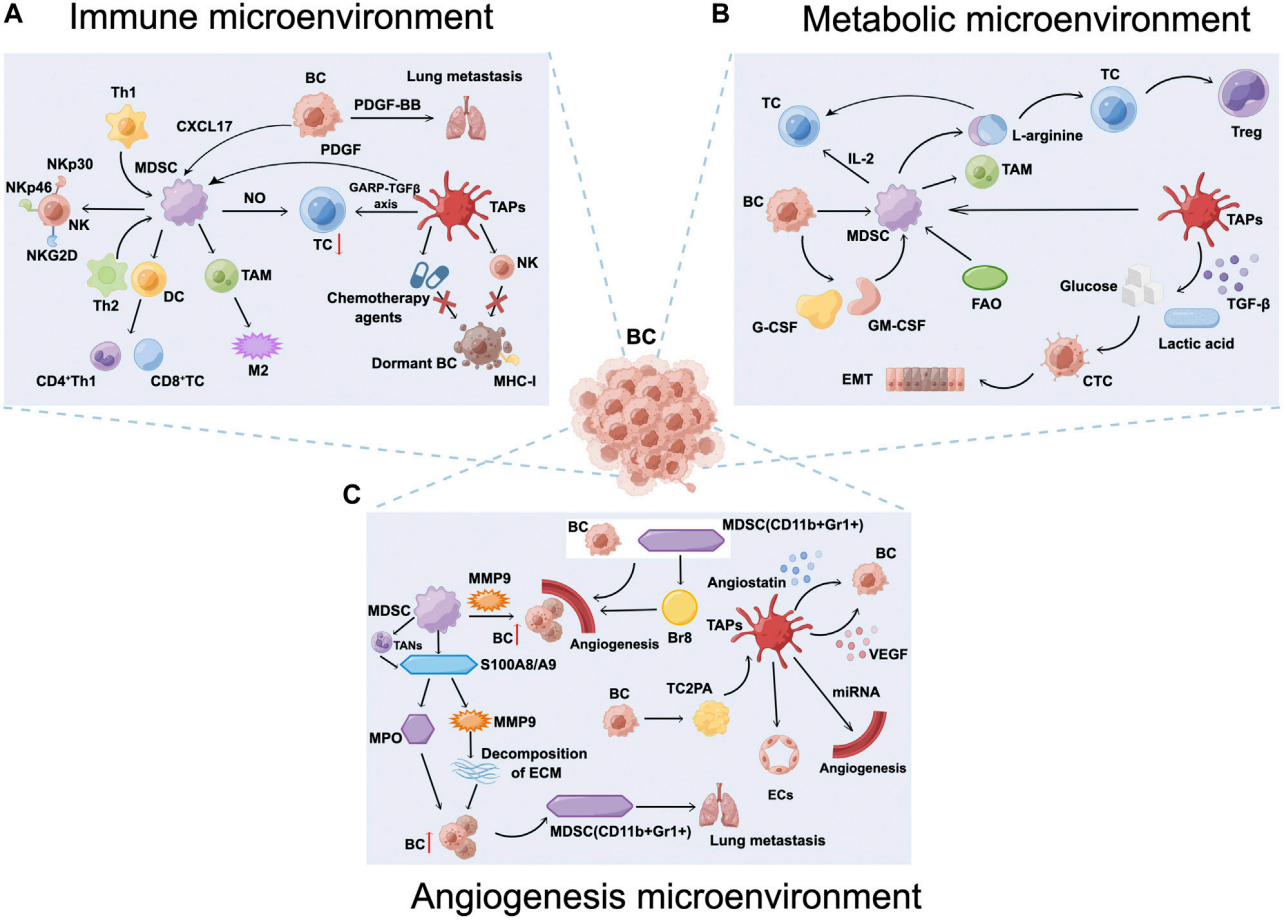
Breast cancer (BC) microenvironment. **(A)** Myeloid-derived suppressor cells (MDSCs) and tumor-associated platelets (TAPs) inhibit the anti-tumor immunity of T and natural killer (NK) cells, secrete interleukin (IL)-10 and TGF-β, induce Treg cells proliferation, and form an immunosuppressive microenvironment that promotes the establishment of BC lung metastases. **(B)** The BC metabolic microenvironment, including abnormalities in glucose metabolism, amino acid metabolism, and lipid metabolism, is a key factor leading to tumor microenvironment immunosuppression as well as tumor immune escape. **(C)** MDSCs and TAPs increase angiogenesis and vascular permeability, induce BC cell dormancy, promote tumor cell extravasation and survival, increase the risk of BC recurrence, participate in BC proliferation, and ultimately promote lung metastasis. Figure was created by Figdraw (www.figdraw.com).

Furthermore, platelets are also associated with T cells, MDSCs, and granulocytes in the BC immune microenvironment. Platelets are the main source of functional TGF-β in the TME. Rachidi et al. (Rachidi et al., 2017) revealed that platelets can limit T cell-mediated anti-tumor immunity through the glycoprotein A repetitions predominant (GARP)-TGF-β axis. Studies have shown that PDGF is associated with the action of MDSCs. Furthermore, BC cells increase the infiltration of MDSCs in the lungs through secretion of CXCL17 and PDGF-BB, which promotes the establishment of lung metastases, leading to BC-associated lung metastasis (Hsu et al., 2019). LaBelle M et al. (Labelle et al., 2014) demonstrated that granulocyte recruitment depends on the secretion of chemokines CXCL5 and CXCL7 by activated platelets, and that chemokines CXCL5 and CXCL7, and blocking this process can effectively prevent the formation of the early PMN and thereby significantly reduce tumor metastasis (Figure 2A).

### 5.2 Metabolic microenvironment

Metabolic reprogramming refers to the ability of tumor cells to alter their energy metabolism and adapt to hypoxic and nutrient-poor microenvironments for rapid growth (Brand et al., 2016). Abnormalities in glucose, amino acid, and lipid metabolism are the main features of metabolic reprogramming, a key factor leading to immunosuppression in the TME and the immune escape of tumors. In recent years, many studies have shown that metabolic abnormalities in BC and its microenvironment are closely related to tumor metastasis.

In normal cells, glucose is maintained in a relatively balanced state. Under hypoxic conditions, glucose is converted to pyruvate and then to lactate., whereas under normal oxygen levels, pyruvate enters the tricarboxylic acid cycle (TCA). Tumor cells do not utilize the mitochondrial oxidative phosphorylation capacity even in the presence of oxygen, but rather utilize aerobic glycolysis, which is defined as the Warburg effect (Vaupel and Multhoff, 2021). Research has shown that glycolysis is involved in tumor development (Gouirand et al., 2018). Tumor cells take up a greatly enhanced amounts of glucose, more than 10 times the amount of glucose taken up by normal cells. Through glycolysis, tumor cells produce large amounts of lactic acid, CO_2_, and other metabolites, which subsequently inhibit effector T cells from exerting anti-tumor effects and inhibit the maturation and activation of DCs. Therefore, tumor cells proliferate under the regulation of the loss of immune T cells and DCs. Clinical studies have shown that the lactate content of tumor tissues is positively correlated with metastasis (Walenta et al., 1997). Experimental studies have confirmed that lactic acid promotes the invasive metastatic potential of BC (Bonuccelli et al., 2010).

A low pH and the accumulation of metabolites in the TME can alter the regulatory processes in MDSCs and platelets. Excess lactate can similarly promote the polarization of MDSCs, and optimal platelet function requires a robust oxidative phosphorylation system. Both aerobic glycolysis and oxidative phosphorylation are enhanced in tumor-associated MDSCs and platelets, compared to those in peripheral MDSCs and platelets (Akahori et al., 1995). As a result of enhanced glycolysis, the expression of lactate dehydrogenase, which facilitates the production of lactate from pyruvate, is also increased, and reduces the expression of lactate dehydrogenase A in tumor cells, leading to a reduction in tumor growth and a decrease in the proportion of splenic MDSCs (Li et al., 2018). Uncontrolled tumor growth causes microenvironmental hypoxia, which not only facilitates tumor invasion and metastasis, but also biases MDSCs toward an immunosuppressive TAM phenotype that is more conducive to immune escape (Corzo et al., 2010). Tumor cell glycolysis, through molecular networks such as AMPK-ULK1, autophagy, and the enhancer binding protein beta (CEBPB) pathway, inhibits the expression of tumor granulocyte colony-stimulating factor (G-CSF) and granulocyte-macrophage colony-stimulating factor (GM-CSF) expression, thereby promoting the proliferation of MDSCs and maintaining tumor immunosuppression (Li et al., 2018). In addition, platelets, which are a major source of TGF-β, can provide a metabolic advantage to CTCs and induce EMT by increasing glucose uptake and lactate secretion (Liu et al., 2016). Therefore, aerobic glycolysis controls the formation of MDSCs (Hu et al., 2020) and consequently influences tumor progression and outcome through distinct molecular pathways.

In tumor cells, the rate of *de novo* fatty acid synthesis is typically enhanced, and at the same time, fatty acid synthesis produces vital cell membrane elements and other significant lipid cellular structures for the proliferation of immune cells, which is essential for immune cell differentiation and functioning. In addition, aberrant long-chain and short-chain fatty acid accumulation and cholesterol exist in immunosuppressive immune cells, such as MDSCs and other immune cell types. MDSCs generally utilize pathways, such as cellular fatty acid oxidation or lipid oxidation, to obtain energy (Pearce and Pearce, 2013), and their mechanism may be to exert immunosuppressive functions through metabolic reprogramming (Hofer et al., 2021). It has been reported that when metabolic reprogramming occurs in MDSCs, mitochondrial fatty acid oxidation (FAO) becomes one of the main energy supply pathways, and the metabolism is enhanced by carnitine palmitoyltransferase 1A (CPT1A) (Hossain et al., 2015). This enables MDSCs to assume enhanced migratory ability and be recruited by tumor-released chemokines into the metastatic region of the tumor (Mohammadpour et al., 2021). CPT1A is a mitochondrial enzyme essential for FAO because it produces acylcarnitines and transports them from the cytoplasmic matrix to the intermembrane lumen of the mitochondria. Several studies have confirmed that FAO is a complementary energy supply for normal organisms during sugar energy deficiency (Joint, 1985). In an energy-deficient TME, the catabolism of fatty acids is a more efficient means of energy supply, especially in immunosuppressed cell populations (Ma and Zhang, 2021).

Reprogrammed amino acid metabolism in BC cells results in a lack of relevant amino acids in the TME, which can compromise the activity of immune effector cells. The second-most abundant nutrient in the cell after glucose is glutamine (Gln), which is the most prevalent non-essential amino acid in the human body. Due to their rapid growth, tumor cells frequently rely on Gln catabolism to provide biosynthetic building blocks, energy sources, and internal environmental homeostasis (Ko et al., 2011). To maintain cell proliferation and immunological responses, activated T cells and macrophages exhibit accelerated Gln metabolism (Liu et al., 2017). Because T cell proliferation, activation, and the secretion of associated cytokines are influenced by the competitive Gln consumption of tumor cells, an immunosuppressive milieu can be created. Simultaneously, Gln increases the proportion of immune cells expressing IL-2 and transferrin receptors, including CD4^+^T lymphocytes and MDSCs(Yaqoob and Calder, 1997), which secrete IL-2 and indirectly inhibit the function of activated T cells.

The semi-essential amino acid Arg, which is transformed into ornithine, urea, nitric oxide, and citrulline by Arg-1 and NOS, respectively, is crucial for controlling the immunological response. Concomitant emotional and mental stress persists after the end of treatment in patients with BC, and the response to norepinephrine (NE) is enhanced, promoting the accumulation of MDSCs. MDSCs express high levels of inducible NOS and Arg-1. Increased and activated Arg-1 levels can deplete L-arginine in the TME, disrupting the local proliferative capacity of T cells and directly suppressing T cells. Furthermore, conditions in the TME are also able to differentiate CD4^+^ T cells into Treg cells, destroying the host immune system and enhancing the immunosuppressive effects of MDSCs to activate tumor dormancy and evade immune system surveillance (Li et al., 2019). In contrast, Arg supplementation stimulates cytotoxicity and effector cytokine production in T cells and NK cells, and significantly enhances the anti-tumor immune response in combination with programmed death-ligand 1 (PD-L1) inhibitors (He et al., 2017) (Figure 2B).

### 5.3 Angiogenic microenvironment

In 1971, Folkman proposed that all tumors are dependent on angiogenesis, and blood vessels not only provide oxygen and nutrients for tumor growth but also provide a pathway for tumor metastasis, which is promoted through the activation of the VEGF signaling axis, that induces tumor cells to infiltrate into the tumor vasculature (Kerbel, 2008). Tumor vascular endothelial cells (VECs) are regulated by multiple cytokines and signaling pathways secreted by tumor cells. For example, tumor cells act on VECs by secreting VEGF, which increases tumor vascular permeability, interstitial hydraulic pressure in tumor tissues, abnormally actives cell proliferation and metastasis, abnormally high expression of angiogenesis-related genes, as well as other pro-angiogenic properties (Schaaf et al., 2019). MDSCs are also capable of deriving TGF-β. Ye et al. (Ye et al., 2015) found that VEGF expression was increased after the pretreatment of 4T1 murine breast cancer cells with intravenous TGF-β1 (Noma et al., 2008). Furthermore, this treatment was able to act on ECs through a paracrine mechanism, leading to tumor microvascular malformations, abnormal morphologic changes in the vascular endothelium, inhibition of apoptosis in ECs, and increased vascular permeability (Bordeleau et al., 2017).

MDSCs directly promote tumor angiogenesis. Importantly, platelets are also major players and promoters of angiogenesis (Liu and Cao, 2016). Co-injection of MDSCs (CD11b^+^Gr 1^+^) into tumors increases intratumoral vascular density, promotes tumor vessel maturation, and reduces necrosis (Yang et al., 2004). MDSCs that infiltrate tumor tissues directly promote tumor growth and angiogenesis by secreting MMP9 and differentiating into ECs. MDSCs (CD11b+Gr 1+) also promote angiogenesis by upregulating the expression of Bv8, a mediator of myeloid cell-dependent tumor vascularization, and the proportion of angiogenesis in the tumors of homozygous mice is significantly reduced by treatment with neutralizing Bv8-specific antibodies (Shojaei et al., 2007). Endothelial progenitor cells (EPCs) are involved in tumor angiogenesis, and platelets can recruit EPCs from the bone marrow and induce EPC differentiation and maturation into ECs arrayed in the neovasculature at early metastatic preeclamptic sites (Langer et al., 2006). Platelets can not only be activated by tumor cell-induced platelet aggregation (TCIPA) to release large amounts of pro-angiogenic factors, but also continuously influence the process of angiogenesis from an early stage by secreting particles, miRNAs, and lipids in the presence of a variety of surface receptors to increase vascular permeability (Wojtukiewicz et al., 2017). In addition, the role of platelets in inducing tumor angiogenesis and maintaining the integrity of the vascular endothelium is dependent on the release of various growth factors and derived substances, such as miRNAs from platelets and platelet-derived microparticles (PMP). Platelet-derived mediators, such as MMP, histamine, 5-hydroxytryptamine, VEGF, hematopoietic growth factor, adenosine triphosphate, and platelet-activating factors, are involved in hydrolyzing the basement membrane and weakening the vascular endothelial barrier. During tumor colonization, platelets upregulate the VEC adhesion molecules, enhancing the arrest and extravasation of tumor cell complexes in the vessel wall (Braun et al., 2021).

Tumor masses that reach a certain size can no longer be supported by normal tissue vasculature, and this will cause tumor cells to become highly dependent on nutrients and oxygen, thereby promoting the recruitment of new blood vessels (Semenza, 2003). However, as the tumor grows to a particular size, it may not be able to draw fresh blood or alter existing arteries (Folkman, 2002). In tumors that are unable to recruit blood vessels, the number of apoptotic cells is equal to the number of proliferating cells. Over time, the two form an equilibrium, resulting in a tumor that cannot expand efficiently (Naumov et al., 2006), thereby causing a state described as angiogenic dormancy. The capacity of tumor cells to respond to hypoxia, promote angiogenesis, and break angiogenic dormancy is known as the angiogenic switch (Aguirre-Ghiso, 2007). Platelets store more than 30 pro-angiogenic or anti-angiogenic regulators (Lal et al., 2013). Vascular dormancy is the result of a balance between pro-angiogenic factors (VEGF and PDGF) and anti-angiogenic factors (thrombospondin, endostatin, and vasopressors) (Naumov et al., 2006), leading to tumor growth inhibition. Therefore, tumors may enter a dormant state if the actively proliferating tumor cell population lacks angiogenesis or hypoxia. Histological analysis (Indraccolo et al., 2009) suggests that the number of ECs in dormant tumors is low and are often located at the margins of the tumor, indicating that these tumors are almost devoid of angiogenesis. Another study (Indraccolo et al., 2009) demonstrated that dormant tumor cells secrete high levels of thrombospondin 1 (TSP1), which has a potent angiogenesis inhibitory effect. Folkman et al. (Folkman, 2006) suggested that tumor cells would enter dormancy if they cannot induce complete tumor angiogenesis. Therefore, the balance between pro-angiogenic and anti-angiogenic factors or the enhancement of anti-vascular factors leads to tumor dormancy.

Outside tumor cells, the ECM prevents dormant cancer cells from activating proliferation. MDSC-derived S100A8/A9 significantly elevates the release of human matrix metalloproteinases from cancer cells to catabolize the ECM (Hu et al., 2021), increasing the invasiveness of proliferative BC tumors (Brisson et al., 2015) and causing widespread recurrence and metastasis (Di Martino et al., 2022). The decreased expression of the EMT-regulated protein Twist1 causes cancer cells to undergo EMT, which promotes tumor recurrence or reactivation by restoring the epithelial features of cancer cells. S100A8/A9 also activates myeloperoxidase (MPO) activation, releases and accumulates oxidized lipids, and upregulates the fibroblast growth factor receptor (FGFR) pathway in tumor cells, causing tumor cells to be released from dormancy, reactivated, and form new tumor foci. In serum samples from 80 patients with surgically treated tumors, researchers found that patients with high serum S100A8/A9 concentrations experienced a significantly shorter period before cancer recurrence (Wagner et al., 2019). In other words, patients with tumors carrying high levels of S100A8/A9 protein were at a higher risk of recurrence. Epidemiological studies have shown that inflammation is associated with a higher risk of BC recurrence after clinical dormancy (Albrengues et al., 2018). The pro-inflammatory mediator S100A8/A9 is involved in promoting inflammatory responses to infections and autoimmune diseases, and has been identified as a potent amplifier of tumor invasion and metastasis (Strupat et al., 2000). S100A8/A9 proteins that accumulate in tumor-bearing mouse serum are secreted by PMN-MDSCs. Meanwhile, S100A8/S100A9 from BC cells binds to receptor for advanced glycation end products on MDSCs and can activate the mitogen-activated protein kinase and nuclear factor kappa-B (NF-κB) signaling pathways in tumor cells to stimulate tumor invasion (Sinha et al., 2008). Neutrophils have different functions in the tumor microenvironment and are classified under different terms, including N1/N2 neutrophils, PMN-MDSCs, and tumor-associated neutrophils (TANs). PMN-MDSCs have a phenotype similar to that of TANs and can be converted to TANs in an immunosuppressive environment. Before recurrent metastasis occurs, new TMEs are not yet formed. However, a large number of TANs arrive at the metastatic location, and the phenotypic transformation of TANs that occurs in PMN-MDSCs recruited by emotional stress such as depression may be one of the key triggers. The inflammatory microenvironment associated with TANs contributes to the pulmonary metastatic colonization of tumor cells. NETosis is the inflammatory cell death mode of TANs accompanied by the formation of neutrophil extracellular traps (NETs) (Steinberg and Grinstein, 2007). This process refers to the release of NETs consisting of depolymerized chromatin and intracellular granule proteins by activated TANs into the extracellular compartment to capture and kill pathogens, and CTCs, which release inflammatory mediators that promote CTCs colonization by establishing an inflammatory environment (Wang et al., 2021) (Figure 2C).

## 6 Treatment

Based on the interactions among MDSCs, TAPs, and BC, many preclinical studies, clinical studies, TCM therapies, and new technologies targeting the improvement of the immune, metabolic, and angiogenic microenvironments of BC through the modulation of MDSCs, TAPs, and BC are underway (Table 1) and provide perspectives on future developments.

**TABLE 1:** Research progress of related treatment.

Classification	Drug	Mechanism	Result	References
**Preclinical studies**	Olaparib	Inhibition of MDSCs recruitment through the SDF1α/CXCR4 axis	Improvement of anti-tumor efficacy of CAR-T cells in mice with BC	Sun et al. (2021)
	Entinostat	Inhibition of MDSCs through activation of STAT3, also drives changes in the phenotype and function of tumor-infiltrating MDSCs and reduces the production of inhibitory factors through the NF-kB and OXPHOS pathways	Improvement of immunosuppressive TME and enhancement of response to ICIs in BC patients	Sidiropoulos et al. (2022)
	Melatonin and Doxorubicin	Immunomodulatory effects by decreasing MDSCs cell expression	Reduction of primary BC tumor growth and distant metastasis	Tanriover et al. (2022)
	Nifuroxazide	Reduction of MDSCs in the lungs of BC mice	Inhibition of BC lung metastasis	Yang et al. (2015)
	Ibrutinib	Induces conversion of MDSCs to DCs, increases the number of more mature DCs, and decreases the number of MDSCs	Reducing tumor burden, inhibiting BC progression and metastasis	Varikuti et al. (2020)
	Sulforaphane	Reduced PGE2 secretion triggered switching of MDSCs to an immunogenic phenotype and enhanced anti-tumor activity of CD8^+^ T cells	Reversing the BC immunosuppressive microenvironment and assisting in improving chemotherapy efficacy	Rong et al. (2020)
	Cabozantinib	Depleting MDSCs	Improved efficacy of anti-HER2 antibody immunotherapy in the 4T1-HER2 mouse BC model	Bakhtiarvand et al. (2022)
	Metformin	Significantly reduced M-MDSCs (CD11b+Gr-1+) and regulatory T cells (Tregs, CD4^+^CD25+Foxp3+), resulting in suppressed expression of the immune checkpoint molecule PD-1 on T cells	Enhanced local antitumor activity in BC TME	Jiang et al. (2023)
	ACT001	Upregulation of 2-associated X protein expression in B-cell lymphoma significantly reduced GM-CSF levels in 4T1 tumors, decreased the number of MDSCs, and inhibited angiogenesis	Induction of apoptosis in 4T1 cells	Liu et al. (2020)
	Aspirin	Inhibit platelet aggregation	Reduced BC invasion and metastasis	Pulcinelli et al. (2004)
	Ticagrelor	Significantly reduced tumor cell-platelet aggregation in the lungs	reduces tumor growth and metastasis	Gareau et al. (2018)
	Tamoxifen	Reduced platelet activation response and reduced vascular endothelial growth factor release	Reduced BC angiogenesis and metastatic potential	Johnson et al. (2017)
**Clinical studies**	(UMIN000022494) propagermanium	Inhibition of PMN formation by decreasing CCL2 and IL-6 concentrations	Inhibition of metastasis in perioperative patients with primary BC	Masuda et al. (2020)
	(NCT01740427 and NCT01942135) CDK4/6 inhibitor palbociclib and endocrine therapy	Significantly inhibited T-cell exhaustion and reduced MDSCs	CDK4/6 inhibitors enhance systemic anti-tumor immunity and are able to reduce patients' dNLR; neutrophil/[leukocyte-neutrophil] values thus predicting a better PFS.	Kim et al. (2023)
	Aspirin	Reversal of platelet activation of Akt signaling pathway in tumor cells leads to decreased IL-8 secretion	Ability to impair BC cell invasion, reduce the incidence of cancer over the age of 3, and enable tumor patients to demonstrate a higher probability of survival	Rothwell et al. (2012)
**TCM**	Ginsenoside Rg3	Inhibition of STAT3-dependent pathways, tumor-derived cytokines and NOTCH signaling pathways	Inhibition of cancer stemness and EMT induced by MDSCs in BC	Song et al. (2020)
	Epigallocatechin-3-gallate	Significantly reduced MDSCs accumulation and increased the proportion of CD4^+^ and CD8^+^ T cells in the spleen and tumor sites of 4T1 BC mice through the Arg-1/iNOS/Nox2/NF-κB/STAT3 signaling pathway	Improvement of BC immunosuppressive microenvironment	Xu et al. (2020)
	Artemisinin	Promoting T cell activation and suppressing immunosuppression of Tregs and MDSCs in tumors	Impedes the growth of 4T1 tumors in the body	Cao et al. (2019)
	α-Hederin	Disruption of PAF/PTAFR Axis Cascade STAT3/MMP-2 Expression	Inhibition of TAPs-activating factor-induced metastasis	Cao et al. (2022)
	Caulis Spatholobi Extract	Reversal of EMT triggered by tumor cell-platelet interaction by blocking platelet-derived PDGF-B release	Significantly inhibited BC invasion and metastasis	Sun et al. (2020)
	Shuang shen granules	Attenuates differentiation of BMCs into MDSCs and reduces CD11b+Ly6C + Ly6G + cells by inhibiting the mTOR/S6K1/Myc signaling pathway	Inhibition of BC lung metastasis	Wei et al. (2021)
	Shugan Jianpi Formula	Reduction of CD8^+^ T-lymphocyte apoptosis and tumor cell activity, enhancement of immune surveillance and inhibition of MDSCs proliferation	Modulation of the BC immunosuppressive microenvironment leading to prolonged survival of tumor-bearing mice	Lu et al. (2017)
	Xuanhusuo San	Reducing the ratio of PMN-MDSC in spleen, decreasing CD11b and Ly6G co-expression in spleen, down-regulating G-CSF mRNA level in 4T1 cells, impeding the differentiation of MDSC to PMN-MDSC through downregulation of G-CSF, and reconstructing the myeloid microenvironment of spleen	Fulfillment of the anti-BC role	Mao et al. (2023)
	Baoyuan Jiedu decoction	Inhibition of TGF-β, Smad2, Smad3, p-Smad2/3, Smad4, and CCL9 protein and gene expression in the TGF-β/CCL9 signaling pathway	Inhibition of MDSCs accumulation in the PMN of lungs	Tian et al. (2020)
**New technologies**	17-AAG	Significantly increased tumor-infiltrating T cells, decreased hypoxia levels, and reduced suppressive lymphocytes in the TME, such as tumor-associated macrophages and MDSCs	Reversing the BC immunosuppressive microenvironment and facilitating checkpoint blockade immunotherapy	Liu et al. (2020)
	LMWH-ATRA	Inhibits the recruitment of MDSCs by competitive binding to PS on the surface of VECs, while hydrophobic segment ATRA promotes the depletion of MDSCs by inducing their differentiation	Significantly improves the inflammatory and immunosuppressive microenvironment at lung and tumor sites and inhibits PMN formation	Lu et al. (2022)
	Nano-DOX	downregulated G-CSF and inhibited the expression and phenotype of MDSCs induced by 4T1 cells	Inhibition of 4T1 cells	Yuan et al. (2019)
	liposomal doxorubicin and liposomal vaccine containing E75 combination treatment	Reduced the expression of MDSCs and the level of ROS, as well as the expression of MDSCs-related genes of Arg1, iNOS, S100A8, and S100A9. Enhanced INF-γ production by immune splenocytes and enhanced the proportion of anti-tumor CD8^+^ and CD4^+^ T cells	Improved therapeutic effects of BC	Zamani et al. (2020)
	FA-CD@PP-CpG synergized with phototherapy (Phototherapy) and docetaxel	Promotes CTL infiltration, inhibits MDSC, and effectively polarizes MDSC toward the M1 phenotype	Enhancing the efficacy of anti-PD-L1 antibodies to enhance immunotherapy against BC	Chen et al. (2019)
	PM-NVs	PM-NVs efficiently accumulate at tumor sites and promote TRAIL interaction with platelet membranes	Significantly inhibited BC progression and reduced lung metastasis	Hu et al. (2015)

### 6.1 Preclinical studies

Preclinical studies can minimize risk by evaluating the various aspects of drugs before using them in human patients and by initially determining the feasibility of a drug or treatment regimen. Olaparib is a PARP inhibitor, which kills tumor cells mainly by engaging in DNA-deficient repair pathways and shows significant potential in BC (Sun et al., 2021). Sun et al. found that Olaparib may inhibit the release of SDF1α in CAFs through HIF1α, reduce the expression of CXCR4, and further limit the recruitment of MDSCs into tumor tissues, and promote the survival of CD8^+^ T cells in order to improve the immunosuppressive microenvironment, thus enhancing the anti-tumor efficacy of Chimeric antigen receptor T cells against mouse BC (Sun et al., 2021). Entinostat not only inhibits MDSCs through the activation of STAT3 but also facilitates changes in the tumor-infiltrating MDSC phenotype and function, and reduces the production of inhibitory factors through the NF-kB and OXPHOS pathways (Sidiropoulos et al., 2022). The combination of Melatonin and Doxorubicin reduces primary tumor growth and distant metastasis, and exerts an immunomodulatory effect by reducing MDSC expression (Tanriover et al., 2022). Nifuroxazide inhibits lung metastasis by decreasing the number of MDSCs in the lungs of BC mice (Yang et al., 2015). By triggering apoptosis, chloroquine can greatly slow the development of 4T1 murine breast cancer cells in culture, inhibiting the secretion of TGF-β, and boosting the immune system through the upregulation of CD8^+^T cells, and the downregulation of TAMs, MDSCs, and Tregs to exert anti-BC effects (Zhang et al., 2018). Ibrutinib prevents BC development and metastasis by inducing the differentiation of MDSCs into DCs. The spleens and tumors of brutinib-treated mice contained more mature DCs and fewer MDSCs, and tumor burden and metastasis were significantly reduced (Varikuti et al., 2020). It has been demonstrated that the cruciferous plant ingredient sulforaphane (SFN), which inhibits and prevents cancer, is effective. Reduced prostaglandin E2 (PGE2) secretion by 4T1 cells treated with SFN causes MDSCs to convert to an immunogenic phenotype, which increases CD8+T cells' anti-tumor activity and reverses the immunosuppressive microenvironment, making it an effective adjuvant chemotherapy candidate for BC (Rong et al., 2020). In a 4T1-HER2 murine BC model, cabozantinib increased the effectiveness of anti-HER2 antibody immunotherapy by depleting MDSCs (Bakhtiarvand et al., 2022). M-MDSCs (CD11b+Gr-1+) and Tregs (CD4^+^CD25+Foxp3+) were significantly reduced in the spleens of mice treated with metformin, and expression of the immune checkpoint molecule PD-1 was suppressed in T cells. Metformin enhances local anti-tumor activity in the TME and is a candidate for BC treatment (Jiang et al., 2023). Secretory granulocyte-macrophage colony-stimulating factor (GM-CSF) promotes angiogenesis and MDSCs proliferation in a dose-dependent manner. The new semiterpene lactone derivative ACT001 possesses anticancer properties and can reduce GM-CSF secretion by inhibiting NF-κB activity in mouse TNBC 4T1 tumors, which in turn, reduces the number of MDSCs and angiogenesis, modulates the tumor microenvironment, and inhibits BC growth and metastasis (Liu et al., 2020).

Aspirin is a well-established platelet inhibitor that reduces BC cells invasion and metastasis (Machlus et al., 2017). The binding of aspirin to COX-1 inhibits platelets aggregation (Pulcinelli et al., 2004). Low-dose aspirin (≤100 mg daily) exerts its anticancer effects by affecting platelets. Platelet activation is primarily mediated by ADP at the P2Y12 receptor on platelets (Tao et al., 2021). Ticagrelor, a P2Y12 inhibitor, is clinically used to prevent cardiovascular and cerebrovascular events and to slow the growth and metastasis of tumors (Gebremeskel et al., 2015). In homozygous mice with *in situ* 4T1 BC, tumor cell-platelet aggregation in the lungs was significantly reduced at 10, 30, and 60 min after treatment with ticagrelor (Gareau et al., 2018). Tamoxifen is a selective modulator of estrogen receptors that is widely used to treat BC. Platelets isolated from patients on tamoxifen maintenance showed a reduced platelet activation response, decreased vascular endothelial growth factor release, and reduced angiogenic and metastatic potential (Johnson et al., 2017).

### 6.2 Clinical studies

Clinical studies are required to evaluate the safety and effectiveness of new treatments for BC. New treatments may lead to better outcomes, but may also carry more risks. Through clinical trials, the safety and efficacy of new treatments can be evaluated to provide better treatment options for patients with BC. Preclinical studies have shown that GB1275 of IL-1 reduces MDSC expression in BC and increases the effectiveness of immunotherapy in BC mouse models. Phase 1/2 clinical research on GB1275 is currently being conducted in patients with advanced solid tumor types (including pancreatic, breast, and prostate cancers) that are known to be resistant to or unlikely to respond to immuno-oncology therapy (NCT04060342) based on encouraging findings from preclinical investigations (DeNardo et al., 2021). The loss of FBXW7 in stromal cells originating from the bone marrow promotes cancer spread by increasing the chemokine CCL2 levels (Yumimoto and Nakayama, 2015). Treatment with propagermanium (PG), a CCL2 inhibitor used to treat chronic hepatitis B in Japan, reduced metastasis in FBXW7-deficient mice by preventing the development of PMN (Masuda et al., 2020). In a phase I dose escalation study, individuals with primary BC undergoing surgery were administered PG as an antimetastatic drug, and blood levels of FBXW7 mRNA were inversely correlated with concentrations of CCL2 and IL-6 in 12 patients administered a maximum dose of 90 mg/body/day PG (UMIN000022494) (Masuda et al., 2020). A multicenter retrospective cohort study and analysis of the PALOMA-2/3 study with immune correlation revealed that treatment of patients with HR-positive, HER2-negative advanced BC with the CDK4/6 inhibitor palbociclib and endocrine therapy significantly suppressed T cell exhaustion and reduced MDSCs to enhance systemic anti-tumor immunity, and was able to improve patient PFS (NCT01740427 and NCT01942135) (Kim et al., 2023).

In a small prospective study of patients administered tamoxifen co-treatment with aspirin was strongly associated with reduced intraplatelet VEGF levels and elevated serum and platelet TSP-1 levels, which were reversed by aspirin discontinuation (Holmes et al., 2013). Johnson et al. (Johnson et al., 2019) demonstrated that aspirin enhances the immunity of tumor cells by reversing the effect of platelets on the activation of the Akt signaling pathway in tumor cells, resulting in less tumor cell invasion and decreased IL-8 secretion. In six trials (35,535 participants) with patients receiving daily low-doses of aspirin for primary prevention, Rothwell et al. (Rothwell et al., 2012) concluded that aspirin reduced the incidence of cancer in patients over the course of 3 years, resulting in patients exhibiting a higher probability of survival.

### 6.3 Traditional chinese medicine (TCM)

Complementary therapies, including TCM, are becoming increasingly popular as a more “natural' approach to achieving health or improving quality of life. Among the elements of TCM, herbs and botanical preparations involve intricate biological processes that may influence a variety of BC development factors, including cell growth and proliferation, apoptosis, interactions between the host and tumor, and immune function and differentiation (Cohen et al., 2002). Ginsenoside Rg3 (Rg3) has been shown to exert anticancer effects in a variety of tumor models, including BC, as well as significant inhibitory effects on MDSCs. Rg3 works primarily by blocking STAT3-dependent pathways, tumor-derived cytokines, and NOTCH signaling pathways, which prevent BC from developing cancer stemness and EMT induced by MDSCs (Song et al., 2020). Paclitaxel-containing ginsenoside Rg3 liposomes achieved a tumor inhibition rate of 90.3% through dual targeting of the TME and cancer cells for drug-resistant cancer therapy. The mechanism of TME remodeling includes inhibition of IL-6/STAT3/p-STAT3 pathway activation, repolarization of M2 macrophages to an anti-tumor M1 phenotype, inhibition of MDSCs, reduction of cellulose-acetafolic and collagen fibers in the TME, and promotion of apoptosis of tumor cells (Zhu et al., 2023). In a 4T1 mouse BC model (Xu et al., 2020), epigallocatechin-3-gallate (EGCG) dramatically reduced the build-up of MDSCs and increased the fraction of CD4^+^ and CD8^+^ T cells in the spleen and tumor sites of mice with 4T1 mammary tumors, both of which helped alleviate immunosuppression. Its major mode of action is the Arg-1/iNOS/Nox2/NF-B/STAT3 signaling pathway; however, it also affects atypical pathways in MDSCs, such as focal adhesion and ECM receptor interactions (Xu et al., 2020). By encouraging T cell activation and reducing the immunosuppression of Tregs and MDSCs in tumors, artemisinin slows the growth of 4T1 tumors *in vivo* (Cao et al., 2019). *a*-Hederin, a triterpenoid saponin, inhibits tumor-associated platelet-activating factor-induced metastasis by disrupting STAT3/MMP-2 expression in the PAF/PTAFR axis cascade (Cao et al., 2022). Caulis Spatholobi extract significantly inhibited tumor invasion and metastasis, and is a new candidate inhibitor for tumor-associated platelet aggregation that blocks the release of platelet-derived PDGF-BB and reverses EMT triggered by tumor cell-platelet interactions (Sun et al., 2020).

By blocking the mTOR/S6K1/Myc signaling pathway, Shuangshen granules inhibit the development of BMCs into MDSCs and decrease the number of CD11b+Ly6C + Ly6G + cells in BC lung metastases (Wei et al., 2021). In mice, immune function can be variably suppressed by depression and tumor growth, and the microenvironment of advanced 4T1 inflammatory BC may be crucial for its pathogenesis (Liu et al., 2021). By lowering CD8^+^ T lymphocyte apoptosis and tumor cell activity, enhancing immune monitoring capacity, and decreasing MDSC proliferation, a Shugan Jianpi formula was able to regulate the immune microenvironment and increase the survival time of tumor-bearing mice (Lu et al., 2017). After Xuanhusuo San intervened with BC in mice, the proportion of spleen PMN-MDSCs decreased, spleen CD11b and Ly6G co-expression decreased, and G-CSF mRNA levels in 4T1 cells were downregulated, which can play an anti-BC role by down-regulating G-CSF, hindering the differentiation of MDSCs into PMN-MDSCs, and reconstructing the splenic myeloid microenvironment (Mao et al., 2023). Baoyuan detoxification soup inhibited PMN formation in BC and suppressed MDSC recruitment. Mechanistically, Baoyuan detoxifying tang inhibits the accumulation of MDSCs in the PMN of the lungs by suppressing the protein and gene expression of TGF-β, Smad2, Smad3, p-Smad2/3, Smad4, and CCL9 in the TGF-β/CCL9 signaling pathway (Tian et al., 2020).

### 6.4 New technologies

Nanoparticles have numerous applications in the treatment of diseases (Newton and Dobrovolskaia, 2022). Nanotechnology in medical settings, referred to as nanomedicine, is being increasingly used for the prevention, diagnosis, and treatment of various diseases, such as cancer (Patra et al., 2018). The creation of various nanomaterials (such as inorganic, polymeric, or lipid nanoparticles) has aided in the stimulation of anti-tumor immunity (Li et al., 2021) and in overcoming some of the drawbacks associated with conventional drug administration (Irvine and Dane, 2020). In addition, nanomaterials combined with other anticancer therapies have the potential to reduce drug resistance in cancer models (Yang et al., 2019). The novel nanomaterial 17-(allylamino)-17-demethoxygeldanamycin (17-AAG), prepared using a thin-film dispersion method, reversed the BC immunosuppressive microenvironment and facilitated checkpoint blockade immunotherapy. Liposome-delivered 17-AAG remodeled the immunosuppressive microenvironment by significantly increasing tumor-infiltrating T cells, decreasing the level of hypoxia, and decreasing suppressive lymphocytes in the TME, such as tumor-associated macrophages and MDSCs (Liu et al., 2020). Micellar nanoparticles, low-molecular-weight-heparin-all-trans-retinoic-acid (LMWH-ATRA), containing the chemotherapeutic drug adriamycin (DOX) and the immunoadjuvant *a*-galactosylceramide by competing on the surface of VECs for PS binding to inhibit MDSCs recruitment. Furthermore, all-trans retinoic acid (ATRA), a hydrophobic molecule, encourages MDSC differentiation and depletion. By modulating MDSCs, micelles can drastically reduce the inflammatory and immunosuppressive microenvironments in the lung and tumor sites, and prevent PMN development (Lu et al., 2022). Doxorubicin-polyglycerol-nanodiamond conjugate (nano-DOX) is a cytostatic inhibitor of 4T1 cells. Nano-DOX downregulates G-CSF and inhibits MDSC expression and the MDSC phenotype induced in 4T1 cells (Yuan et al., 2019). Combination treatment with doxorubicin liposomes and an E75-containing liposomal vaccine reduced MDSC expression and ROS levels, as well as Arg1, iNOS, S100A8, and S100A9 expression associated with MDSCs. Enhanced immune splenocytes produced INF-γ and enhanced the ratio of anti-tumor CD8^+^ and CD4^+^ T cells, which improved the therapeutic efficacy of BC treatments (Zamani et al., 2020).

The novel FA-CD@PP-CpG nanocomposite synergized with phototherapy and docetaxel to enhance its immunotherapeutic effect on BC. Low-dose loading of DTX in FA-CD@PP-CpG promoted cytotoxic T lymphocyte (CTL) infiltration to enhance the efficacy of the anti-PD-L1 antibody to inhibit MDSCs and effectively polarize MDSCs toward the M1 phenotype, further improving anti-tumor efficacy (Chen et al., 2019). Platelet membrane (PM)-coated core-shell nanovehicles (PM-NV) consist of a Dox-loaded nanogel core and a platelet membrane-based shell chemically linked to TRAIL. PM-NVs can effectively accumulate at the tumor site due to the affinity between PS on the platelet membrane and the CD44 receptor overexpressed on cancer cells. PS on the platelet membrane surface mediates the accumulation of PM-NVs, thereby promoting the interaction of TRAIL with the membrane. PM-NVs significantly inhibit BC progression and reduce lung metastasis (Hu et al., 2015).

## 7 Summary and outlook

MDSCs, which are key immunosuppressive cells in the TME, are considered novel targets for anti-tumor immunotherapy and are receiving increasing attention with regards to tumor development and treatment. In recent years, there has been increasing evidence that TAPs help cancer cells evade the immune system and play a role in the metastatic process. Therefore, TAP-targeting strategies have great potential when combined with immunotherapy. Platelets play an important role in blood clot hemostasis through progressive adhesion, activation, and aggregation. Cancer is a non-healing wound that can continuously activate platelets (Menter et al., 2017). By understanding the physiological and pathological characteristics of platelets and their various functions in the emergence of cancer, as well as the interactions between MDSCs and TAPs, including their generation, secretion, activation, and recruitment, we can develop drugs and approaches that target and prevent TAPs from interacting with MDSCs, which may have great potential as cancer therapeutic candidates.

However, due to the diverse MDSC phenotypes, extremely high heterogeneity, complex origins, and functional networks, the therapeutic approaches currently used to treat MDSCs are only partially successful. To develop new clinical targets and strategies, it is necessary to address the complexity and heterogeneity of MDSCs. In addition, the systemic use of antiplatelet agents carries serious complications, and to date, none of the approved antiplatelet agents have completely negated the risk of bleeding. Therefore, combining antiplatelet strategies with other therapeutic approaches could minimize the occurrence of possible side effects.

## Figures and Tables

**Table:** 

**17-AAG**	17-(allylamino)-17- demethoxygeldanamycin
**Arg-1**	Arginase-1
**ATRA**	all-trans retinoic acid
**BC**	breast cancer
**BMDCs**	bone marrow-derived cells
**CP**	cancer procoagulant
**CPT1A**	carnitine palmitoyltransferase 1A
**CTCs**	circulating tumor cells
**CTL**	cytotoxic T lymphocyte
**CXCL17**	C-X-C motif chemokine ligand 17
**DFS**	Disease-free-survival
**ECM**	extracellular matrix
**EC**	endothelial cell
**EGCG**	epigallocatechin-3-gallate
**e-MDSCs**	early stage MDSCs
**EMT**	epithelial-mesenchymal transition
**EPCs**	Endothelial progenitor cells
**ER**	estrogen receptor
**FAO**	fatty acid oxidation
**GARP**	glycoprotein A repetitions predominant
**G-CSF**	granulocyte colonystimulating factor
**GITRL**	glucocorticoid-induced tumor necrosis factor receptor ligand
**GLOBOCAN**	International Agency for Research on Cancer
**Gln**	Glutamine
**GM-CSF**	granulocyte-macrophage colony-stimulating factor
**G-MDSC**	granulocyte-MDSC
**HER2**	human epidermal growth factor receptor 2
**IFN**	interferon
**IL**	interleukin
**iNOS**	inducible NOS
**ITP**	immune thrombocytopenia
**MDSCs**	Myeloid-derived suppressor cells
**MHC**	major histocompatibility complex
**M-MDSC**	monocytic-MDSC
**MMP9**	Matrix metalloproteinase 9
**mTOR**	mammalian target of rapamycin
**nano-DOX**	doxorubicin-polyglycerol-nanodiamond conjugate
**NETs**	neutrophil extracellular traps
**NF-κB**	nuclear factor kappa-B
**NK**	natural killer
**NO**	nitric oxide
**OS**	overall survival
**PDGF-BB**	Platelet-derived growth factor-BB
**PD-L1**	programmed death-ligand 1
**PG**	propagermanium
**PGE2**	prostaglandin E2
**PLR**	platelet-to-lymphocyte ratios
**PM**	Platelet membrane
**PMN**	pre-metastatic niche
**PMN-MDSC**	polymorphonuclear-MDSC
**PR**	progesterone receptor
**PSGL-1**	pselectin glycoprotein ligand-1
**ROS**	reactive oxygen species
**SFN**	Sulforaphane
**TAM**	tumor associated macrophages
**TANs**	tumor-AssociAted neutrophils
**TAPs**	Tumor-associated platelets
**TCA**	tricarboxylic acid cycle
**TCIPA**	tumor cell induced platelet aggregation
**TCM**	traditional Chinese medicine
**TGF-β**	transforming growth factor-β
**TF**	Tissue Factor
**TME**	tumor microenvironment
**TNF-α**	tumor necrosis factor *a*
**Tregs**	regulatory T cells
**TSP1**	thrombospondin 1
**VECs**	vascular endothelial cells
**VEGF**	vascular endothelial growth factor
